# Identification of the Functional Domains of the Telomere Protein Rap1 in *Schizosaccharomyces pombe*

**DOI:** 10.1371/journal.pone.0049151

**Published:** 2012-11-02

**Authors:** Ikumi Fujita, Makiko Tanaka, Junko Kanoh

**Affiliations:** Institute for Protein Research, Osaka University, Suita, Osaka, Japan; Tulane University Health Sciences Center, United States of America

## Abstract

The telomere at the end of a linear chromosome plays crucial roles in genome stability. In the fission yeast *Schizosaccharomyces pombe*, the Rap1 protein, one of the central players at the telomeres, associates with multiple proteins to regulate various telomere functions, such as the maintenance of telomere DNA length, telomere end protection, maintenance of telomere heterochromatin, and telomere clustering in meiosis. The molecular bases of the interactions between Rap1 and its partners, however, remain largely unknown. Here, we describe the identification of the interaction domains of Rap1 with its partners. The Bqt1/Bqt2 complex, which is required for normal meiotic progression, Poz1, which is required for telomere length control, and Taz1, which is required for the recruitment of Rap1 to telomeres, bind to distinct domains in the C-terminal half of Rap1. Intriguingly, analyses of a series of deletion mutants for *rap1* ^+^ have revealed that the long N-terminal region (1–456 a.a. [amino acids]) of Rap1 (full length: 693 a.a.) is not required for telomere DNA length control, telomere end protection, and telomere gene silencing, whereas the C-terminal region (457–693 a.a.) containing Poz1- and Taz1-binding domains plays important roles in those functions. Furthermore, the Bqt1/Bqt2- and Taz1-binding domains are essential for normal spore formation after meiosis. Our results suggest that the C-terminal half of Rap1 is critical for the primary telomere functions, whereas the N-terminal region containing the BRCT (BRCA1 C-terminus) and Myb domains, which are evolutionally conserved among the Rap1 family proteins, does not play a major role at the telomeres.

## Introduction

The telomere is the specialized chromatin structure located at the ends of eukaryotic linear chromosomes. The telomere consists of telomeric DNA containing repetitive sequences (TTAGGG in vertebrates and TTACAG_2–5_ in fission yeast) and various associated proteins [1], [2], [3], [4]. Telomere-binding proteins protect chromosome ends from enzymatic degradation, inappropriate homologous recombination and end-to-end fusion, and regulate the access of telomerase, the telomere-specific reverse transcriptase, to the single-stranded telomere DNA at the chromosome end to maintain the telomere DNA length [5], [6]. Moreover, telomeres play critical roles in the dynamics of meiotic chromosomes [7].

**Figure 1:**
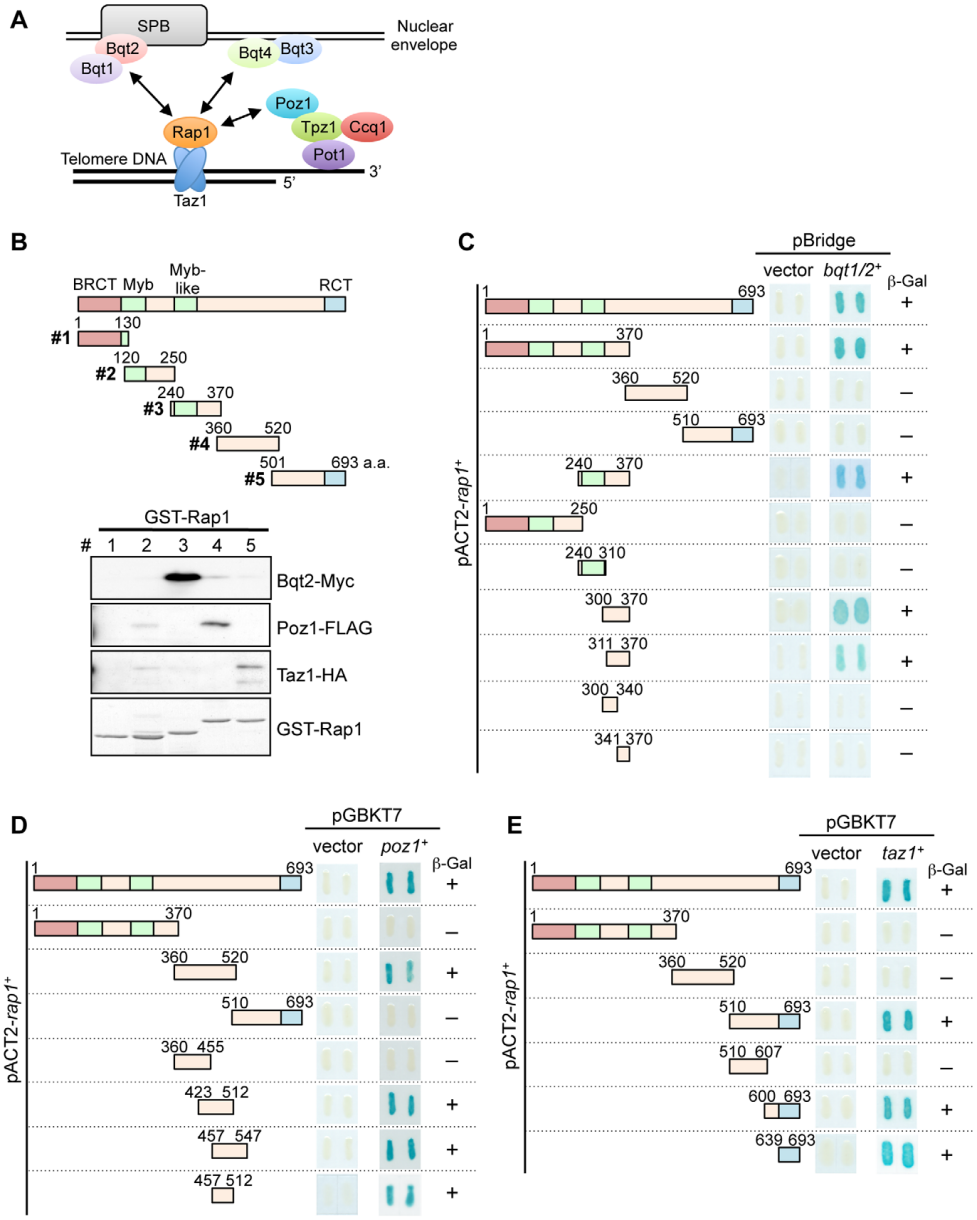
Identification of the Bqt1/Bqt2-, Poz1- and Taz1-binding domains in Rap1. (A) Schematic illustration of the Rap1-binding proteins in *S. pombe*. Arrows indicate the physical interactions. Poz1 also interacts with Tpz1 on the single-stranded telomere DNA. Bqt3 is a receptor for Bqt4 on the inner nuclear membrane. SPB, spindle pole body. (B) Pull-down assays to identify the binding regions of Rap1 with Bqt2, Poz1, or Taz1. A series of GST-Rap1 fusion proteins (#1, 1–130 a.a.; #2, 120–250 a.a.; #3, 240–370 a.a.; #4, 360–520 a.a.; #5, 501–693 a.a.) were purified from bacteria. Each GST-Rap1 protein was mixed with *S. pombe* cell extracts containing Bqt2-Myc, Poz1-Flag, and Taz1-HA. Glutathine bead-bound proteins were separated by SDS-PAGE and detected with anti-Myc, anti-FLAG, and anti-HA antibodies. (C) Three-hybrid assays to detect the interactions between Rap1 and Bqt1/Bqt2. The *rap1* ^+^ DNA fragments (indicated by boxes) were inserted into the pACT2 vector (prey). pBridge-*bqt1* ^+^/*bqt2* ^+^ was used as the bait. The interactions between Rap1 and Bqt1/Bqt2 were assessed by ß-galactosidase activity (indicated by + or –). (D, E) Two-hybrid assays to detect the interactions between Rap1 and Poz1 (D) or Taz1 (E). The *rap1* ^+^ DNA fragments (indicated by boxes) were inserted into pACT2 (prey), and *poz1* ^+^ or *taz1* ^+^ was inserted into pGBKT7 (bait). The interactions were assessed by ß-galactosidase activity (indicated by + or –).

In fission yeast, *Schizosaccharomyces pombe*, the Taz1 protein (a homolog of mammalian TRF1/TRF2) directly binds to the double-stranded telomere DNA [8]. Taz1 recruits the Rap1 protein (a homolog of mammalian Rap1) to the telomeres, and Rap1 in turn associates with multiple proteins to regulate a variety of telomere functions [9], [10], [11], [12], [13], [14], [15] (Figure 1A). For example, Rap1 interacts with Poz1 to form the shelterin complex [16] to negatively regulate the access of telomerase to the chromosome end, the Bqt1/Bqt2 complex to relocate telomeres to the spindle pole body (the equivalent of the centrosome) to form a bouquet structure in meiotic prophase, and Bqt4 to tether telomeres to the inner nuclear membrane during interphase [15], [17], [18]. These observations indicate that Rap1 is the key player that performs the multiple telomere functions through interactions with its various partners.

Rap1 family proteins in eukaryotes contain a BRCT domain, one or two Myb domains, and an RCT (Rap1 C-terminal) domain in common [19]. The two Myb domains of budding yeast Rap1 mediate the direct binding to the double-stranded telomere DNA [20]. The functions of the Myb domains of fission yeast and mammalian Rap1, however, remain largely unknown. Recently, we have shown that the RCT domains of Rap1 in multiple organisms act as a protein-protein interaction module [19]. The direct telomere DNA-binding proteins, Taz1 and TRF2, interact with the RCT domains of Rap1 to recruit Rap1 to telomeres in fission yeast and mammals. In contrast, the RCT domain of budding yeast Rap1 recruits two groups of proteins, the Rif proteins (Rif1 and Rif2) to regulate telomere DNA length and the Sir proteins (Sir3 and Sir4) to mediate gene silencing at telomeres [21], [22], [23], [24]. Although the nature of the RCT domain has been considerably clarified, the molecular functions of the other parts of Rap1 especially in fission yeast and mammals remain largely unknown.

To investigate the molecular bases of the functions of fission yeast Rap1, we identified the domains required for the interactions with its multiple partners and analyzed the effects of various deletions of the coding region of *rap1* ^+^ on the telomere functions. We found that the already known Rap1 partners (Bqt1/Bqt2, Poz1 and Taz1) interact with distinct domains in the C-terminal half of the Rap1 protein. Deletions and a mutation at the C-terminus of Rap1 conferred telomere dysfunctions to cells. In contrast, deletions of the Rap1 N-terminal region containing the BRCT and Myb domains caused no defect in the telomere functions. Our results indicate that the C-terminal half is critical for the primary functions of the telomere.

## Results

### Identification of the interaction domains in Rap1 for its partners

*S. pombe* Rap1 contains three domains, the BRCT, Myb (and Myb-like) and RCT domains, which are conserved among the Rap1 family proteins [12], [19] (Figure 1B). To identify the interaction domains in *S. pombe* Rap1 with its partners, we first performed pull-down assays. The coding region of the *rap1* ^+^ gene was divided into five regions (#1– #5, Figure 1B), and the corresponding GST-Rap1 fusion proteins were purified from *E. coli*. Each GST-Rap1 protein was incubated with the *S. pombe* cell extracts containing Bqt2-Myc, Poz1-Flag, or Taz1-HA proteins. Bqt2-Myc, Poz1-Flag, and Taz1-HA were co-purified with GST-Rap1 #3, #4, and #5, respectively, indicating that they associate with these distinct domains in the C-terminal region of Rap1 (Figure 1B).

Yeast two- or three-hybrid assays were performed to narrow each interaction domain. We found that the Bqt1-Bqt2 complex interacts with 311–370 a.a. of Rap1, and that Poz1 interacts with 457–512 a.a. of Rap1 (Figures 1C and 1D). We also confirmed that Taz1 binds to the most C-terminal RCT domain (639–693 a.a.), as previously reported [19] (Figure 1E).

### Expression of Rap1 variants

**Figure 2:**
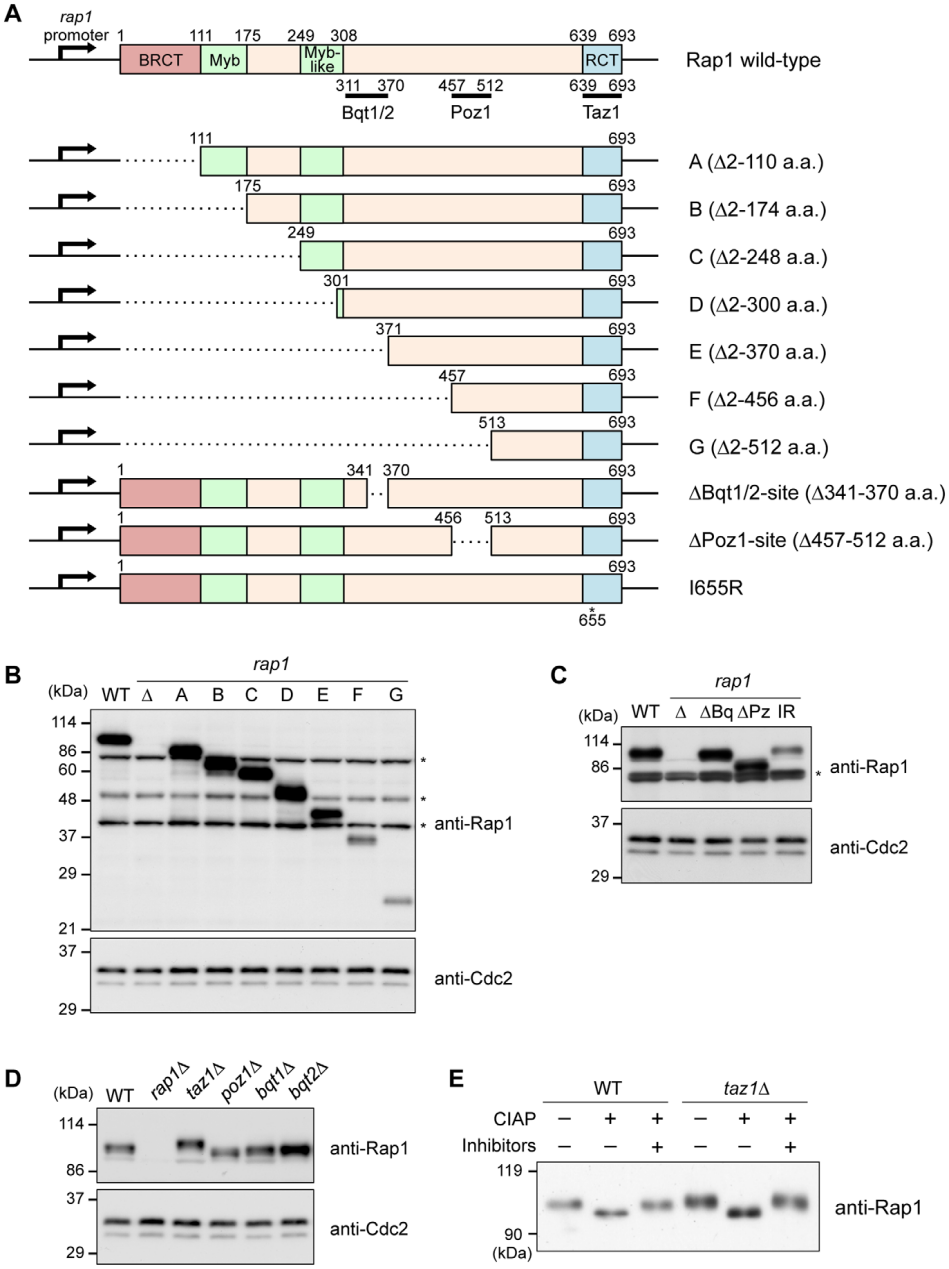
Expression of the Rap1 proteins in various mutants. (A) Schematics of a series of *rap1* deletions at the *rap1* ^+^ locus. The binding sites for Bqt1/Bqt2, Poz1, and Taz1 are indicated by black bars below the Rap1 wild-type. In the *rap1-I655R* mutant, the Ile655 residue was mutated to Arg. (B) Expression of the Rap1 proteins. The whole cell extracts from asynchronous cells were analyzed by immunoblotting using anti-Rap1 antibodies. WT, wild-type; Δ, *rap1*Δ. Asterisks indicate non-specific bands. Cdc2: loading control. (C) Expression of the Rap1 proteins in each strain was analyzed by immunoblotting using anti-Rap1. ΔBq, Bqt1/2-binding site deletion; ΔPz, Poz1-binding site deletion; IR, *rap1-I655R*. (D) Expression of the full-length Rap1 proteins in the various gene deletion mutants. The whole cell extracts of each strain were analyzed by immunoblotting using anti-Rap1. (E) Rap1 is highly phosphorylated in *taz1*Δ. Cells were grown to mid-log phase. Rap1 proteins were immunoprecipitated from the cell extracts using anti-Rap1 antibodies and treated with CIAP for 1 h at 32°C with or without phosphatase inhibitors. The samples were analyzed by immunoblotting using anti-Rap1.

To analyze the physiological importance of each Rap1 domain, series of deletion strains that lacked various N-terminal (*rap1-A* – *-G*) and intermediate regions (*rap1-*Δ*Bq*, the deletion of a part of the Bqt1/Bqt2-binding site [341–370 a.a.]; *rap1-*Δ*Pz*, the deletion of the Poz1-binding site [457–512 a.a.]) were constructed (Figure 2A). We tried to adjust the expression level of each Rap1 variant to that of the wild-type (i.e., full-length) Rap1 because deletion of the full-length Rap1 results in severe defects in the telomere functions [10], [12], [14] and therefore a significant decrease in the Rap1 expression level possibly causes its dysfunction. To adjust the each Rap1 expression level as much as possible, the mutated *rap1* genes were integrated at the genomic *rap1* ^+^ locus with its original promoter and terminator, and we avoided epitope-tagging at the C-terminus because we found that tagging at the C-terminus causes a remarkable change in the expression level of the Rap1 protein (data not shown). Furthermore, only a part of the Bqt1/Bqt2-binding site was deleted in the *rap1*-Δ*Bq* strain because the deletion of the whole Bqt1/Bqt2-binding site caused a marked decrease in the Rap1 protein level (data not shown). Moreover, deletion of the Taz1-binding site also resulted in a striking decrease in the Rap1 protein level (data not shown). Therefore, the *rap1-I655R* strain, in which the isoleucine-655 residue of Rap1 was changed to arginine, was used instead. The Rap1-I655R protein does not interacts with Taz1, thereby inducing an abnormal elongation of telomere DNA and de-protection of chromosome ends, as shown in a previous study [19].

The expression of the Rap variant in each strain was analyzed by western blotting using anti-Rap1 antibodies that recognize the C-terminal region (370–693 a.a.). Strains *rap1-A* to *rap1-E* expressed each Rap1 variant at levels comparable to that of the wild-type (Figure 2B). Strains *rap1-F*, *rap1-G* and *rap1-*Δ*Pz* showed weaker bands of Rap1 variants, likely because the Rap1-F, Rap1-G and Rap1-ΔPz proteins contain smaller antigen-recognition sites for the anti-Rap1 antibody and/or these proteins are expressed at lower levels compared with the full-length Rap1 (Figures 2B and 2C). We also found that the *rap1-I655R* mutation caused a moderate decrease in the level of the Rap1 protein, suggesting that the Taz1-binding site is important for the stability of the Rap1 protein (Figure****2C).

We also analyzed the expression of the full-length Rap1 protein in the strains with deletions of the various Rap1-interactor genes (Figure 2D). None of the gene deletion strains showed an apparent decrease in the full-length Rap1 protein level. Intriguingly, the *taz1*Δ strain showed an upshift of the Rap1 band, whereas *poz1*Δ showed a slight downshift, indicating that Taz1 and Poz1 affect the post-translational modification of Rap1. To investigate the nature of the Rap1 modification in *taz1*Δ, the full-length Rap1 proteins derived from the wild-type and *taz1*Δ strains were treated with calf intestinal alkaline phosphatase (CIAP) (Figure 2E). The phosphatase treatment of Rap1 in both strains caused a marked downshift of the Rap1 bands, indicating that Rap1 is phosphorylated, especially in *taz1*Δ. We have recently shown that Rap1 is hyperphosphorylated at M phase [25]. The increased level of Rap1 phosphorylation in *taz1*Δ may be caused by the possible prolongation of M phase in *taz1*Δ or by other currently unknown mechanisms.

### The Poz1- and Taz1-binding sites are essential for telomere length control

**Figure 3:**
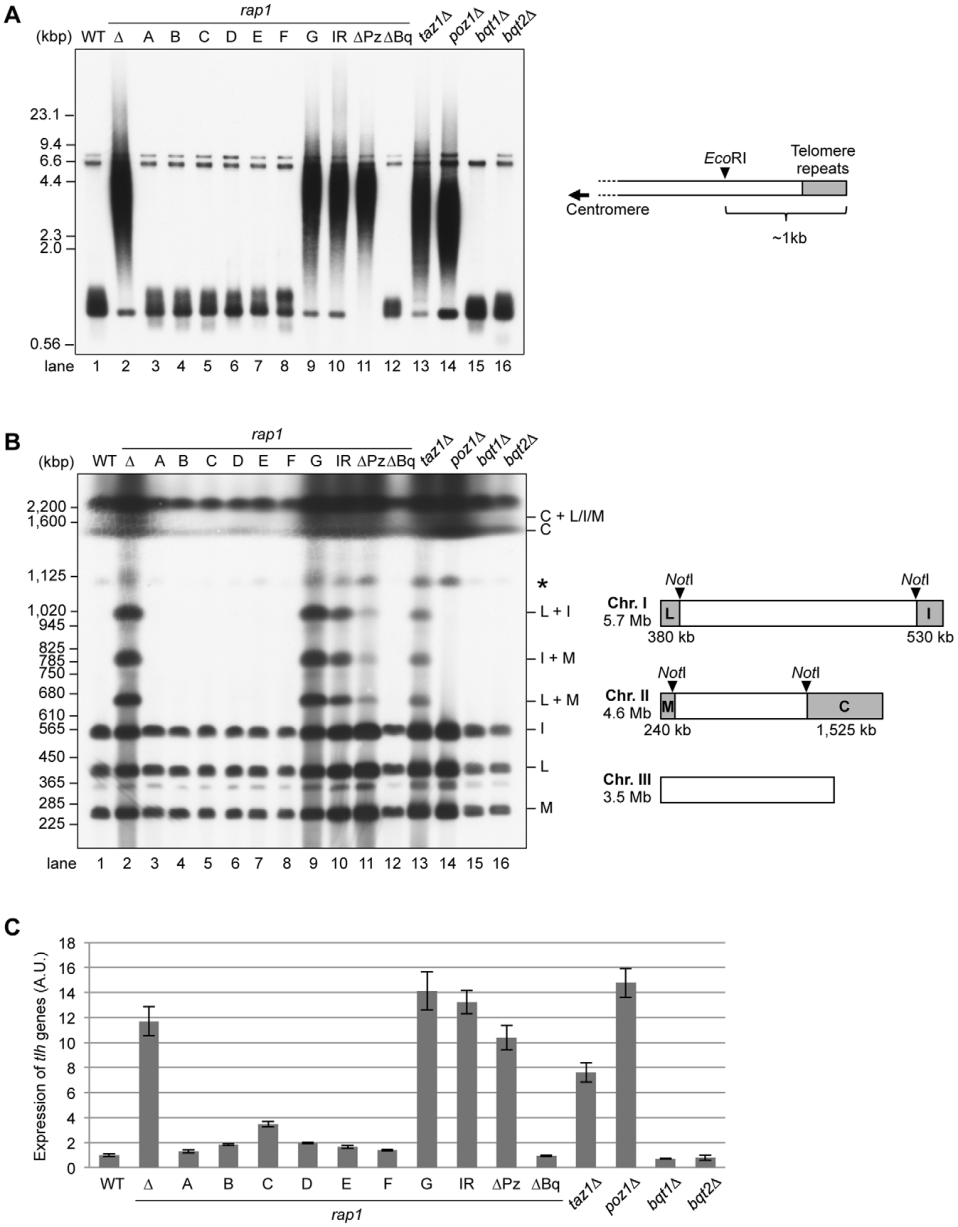
The Poz1- and Taz1-binding sites are critical for the maintenance of telomere structure. (A) Southern blot analysis of the telomere DNA length. Genomic DNA was extracted from each strain grown in YES, digested by the restriction enzyme *Eco*RI, and separated on a TAE-1% agarose gel. The *Eco*RI digestion generates ∼1-kb fragments containing the telomere repeats from chromosomes I and II in the wild-type (right). A probe specific for the telomere repeats was used to detect the telomere ends. ΔBq, Bqt1/2-binding site deletion; ΔPz, Poz1-binding site deletion; IR, *rap1-I655R*. These data were reproduced using the other strains carrying the same *rap1* alleles (Figure S1A). (B) Telomere end protection in G_1_-arrested cells. The strains used in (A) were grown in EMM, shifted into EMM–N (without nitrogen), and incubated at 28°C for 24 hours. Chromosomal DNA was prepared in agarose plugs and separated by PFGE after *Not*I digestion. The gel was transferred to a nylon membrane and hybridized with a probe specific for telomere repeats. The letters on the right side of the southern blot indicate the identities of the *Not*I-digested chromosomal DNA fragments. An asterisk indicates the bands, which probably correspond to the incompletely digested DNA fragment containing the “L” fragment of the chromosome I. (C) Telomere gene silencing. RNA expression level of sub-telomeric *tlh* genes was analyzed by reverse transcription-quantitative PCR. The value of the *tlh* genes was normalized by that of the *his1* gene. Bars and error bars indicate mean and SEM of three or four experiments. A.U., arbitrary units.

To investigate the physiological importance of each Rap1 domain, we analyzed the phenotypes of each *rap1* mutant. Rap1, Taz1, and Poz1 are the key negative regulators of telomerase [8], [10], [12], [18]; thus, deletions of the entire *rap1* ^+^, *taz1* ^+^, or *poz1* ^+^ genes result in a marked elongation of the telomere DNA (Figures 3A, lanes 1–2, 13–14, and S1A). The N-terminal deletion strains *rap1-A* – *-F* did not show any significant change in telomere DNA length, whereas the *rap1-G* mutant exhibited a striking elongation of telomere DNA similar to that in the complete *rap1* deletion strain (Figure 3A, lanes 3–9). The *rap1-*Δ*Pz* and *rap1-I655R* mutations but not the *rap1-*Δ*Bq* also caused a massive elongation of telomere DNA [19] (Figure 3A, lanes 10–12). These results indicated that the Poz1- and Taz1-binding sites are crucial for telomere length control, whereas the N-terminal region (1–456 a.a.) is not required for it.

### The C-terminal region (457–693 a.a.) is sufficient for telomere end protection in the G_1_ phase

To analyze the involvement of each Rap1 domain in the protection of telomere ends, we next examined the NHEJ (non-homologous end joining)-dependent telomere end fusions in G_1_ phase when homologous recombination is inhibited [5]. Because Rap1 and Taz1 are essential for the prevention of the fusion of telomere ends in G_1_ [14], deletions of the *rap1* ^+^ or *taz1* ^+^ genes and the *rap1-I655R* mutation resulted in telomere end fusions, as detected by pulse-field gel electrophoresis (PFGE) of *Not*I-digested chromosomal DNA followed by Southern blotting using telomere probes [19] (Figure 3B, lanes 1–2, 10, 13). Similar to the results displayed in Figure 3A, the N-terminal deletion strains *rap1-A* – *-F* did not show any telomere fusion bands, whereas the *rap1-G* mutant exhibited telomere fusion bands as in the complete *rap1* deletion strain (Figure 3B, lanes 3–9), indicating that the C-terminal region (457–693 a.a.) is sufficient for telomere end protection in G_1_.

The *rap1*-Δ*Bq*, *bqt1*Δ, and *bqt2*Δ mutants did not exhibit telomere end fusions, indicating that the interaction between Rap1 and Bqt1/Bqt2 is not required for telomere end protection in G_1_ (Figure 3B, lanes 12, 15, 16). The *rap1*-Δ*Pz* mutant showed telomere fusions with a lower frequency, as the intensity of the telomere fusion bands was considerably weaker than that in the *rap1*Δ strain (Figure 3B, lanes 2, 11). In contrast, the *poz1Δ* strain did not exhibit any telomere fusion (Figure****3B, lane 14). These data suggest that the deletion of the Poz1-binding site partially compromised the binding of other factors and led to telomere fusion independently of Poz1. The higher frequency of telomere end fusion in the *rap1-G* mutant than in the *rap1-*Δ*Pz* mutant implies that the expression level of Rap1 is also important for the telomere end protection (note that the expression level of Rap1-G is possibly lower than that of Rap1-ΔPz [Figures 2B and 2C]).

### The Poz1- and Taz1-binding sites are essential for the telomere gene silencing

Taz1 and Rap1 are required for the maintenance of telomere heterochromatin structure, and therefore the *taz1*Δ and *rap1*Δ mutants display defects in gene silencing at telomere [8], [12], [26]. To assess the telomere gene silencing in each *rap1* mutant, we analyzed the RNA expression of the *tlh* genes at the distal end of subtelomeres by reverse transcription-quantitative PCR [26](Figures 3C). While the transcription of the *tlh* genes was repressed in the *rap1-A – F*, *rap1-*Δ*Bq*, *bqt1*Δ, and *bqt2*Δ strains as in the wild type, it was highly de-repressed in the *rap1-G*, *rap1-I655R*, *rap1-ΔPz*, *rap1*Δ, *taz1*Δ, and *poz1*Δ strains. These data indicate that the Poz1- and Taz1-binding sites of Rap1 are essential for the subtelomeric gene silencing.

### The Bqt1/Bqt2- and Taz1-binding sites are essential for the normal progression of meiosis

**Figure 4:**
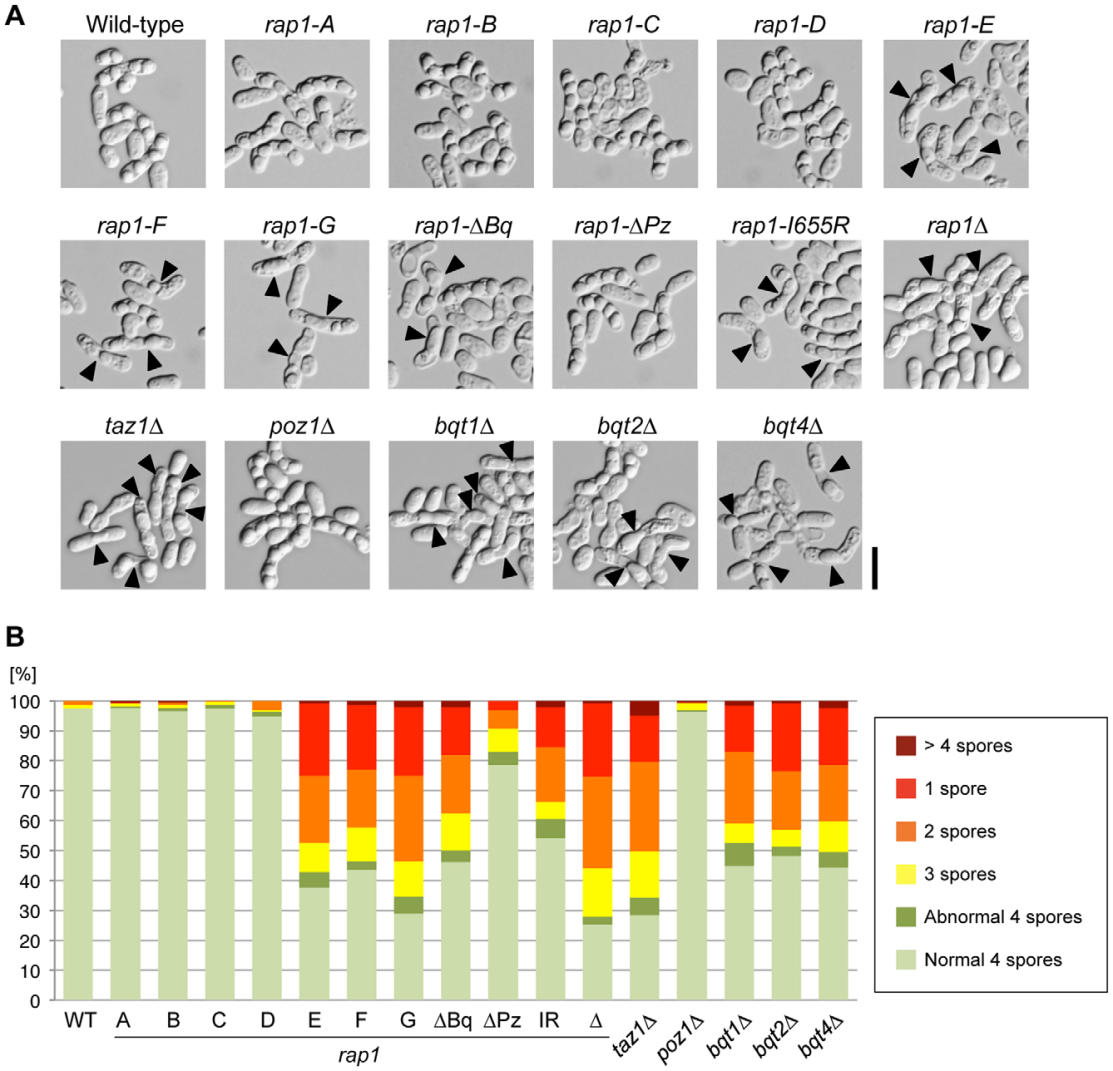
The Bqt1/Bqt2- and Taz1-binding sites are critical for normal spore formation. (A) Homothallic haploid strains were grown on a YES plate and then spotted onto an MEA plate, and incubated at 28°C for 36–38 hours to induce meiosis and sporulation. DIC images are shown. Arrowheads indicate abnormal spores. Bar, 10 µm. (B) Frequency of irregular ascospore formation in each strain under the same conditions as in (A). *n*>200 for each strain. These data were reproduced using the other strains carrying the same *rap1* alleles (Figure S1B).

Rap1 and Taz1 are required for the clustering of the telomeres towards the spindle pole body (SPB) during the meiotic prophase, which is critical for the proper meiotic chromosome segregation and the normal spore formation [9], [10], [12]. Bqt1 and Bqt2 are expressed specifically during meiosis and required for the recruitment of Rap1 with Taz1 and telomere DNA to the SPB [17]. Therefore, the *rap1*Δ, *taz1*Δ, *bqt1*Δ, or *bqt2*Δ strains exhibit asci with an irregular number and morphology of spores after meiosis (Figure****4). We found that Poz1 was dispensable for the normal progression of meiosis because the *poz1*Δ strain did not show a remarkable defect in spore formation. To investigate the importance of each Rap1 domain in meiosis, the spore formation of each *rap1* mutant was examined (Figures 4 and S1B). The N-terminal deletion strains *rap1-A* – *-D* mostly produced four normal spores, whereas the *rap1-E* – *-G* mutants exhibited a high frequency of abnormal spore formation. The *rap1-*Δ*Bq* and *rap1-I655R* mutants also displayed a severe defect in spore formation, whereas the *rap1-*Δ*Pz* mutant only showed a moderate defect. These data suggested that the Bqt1/Bqt2- and Taz1-binding sites are critical for the normal progression of meiosis.

## Discussion

**Figure 5:**
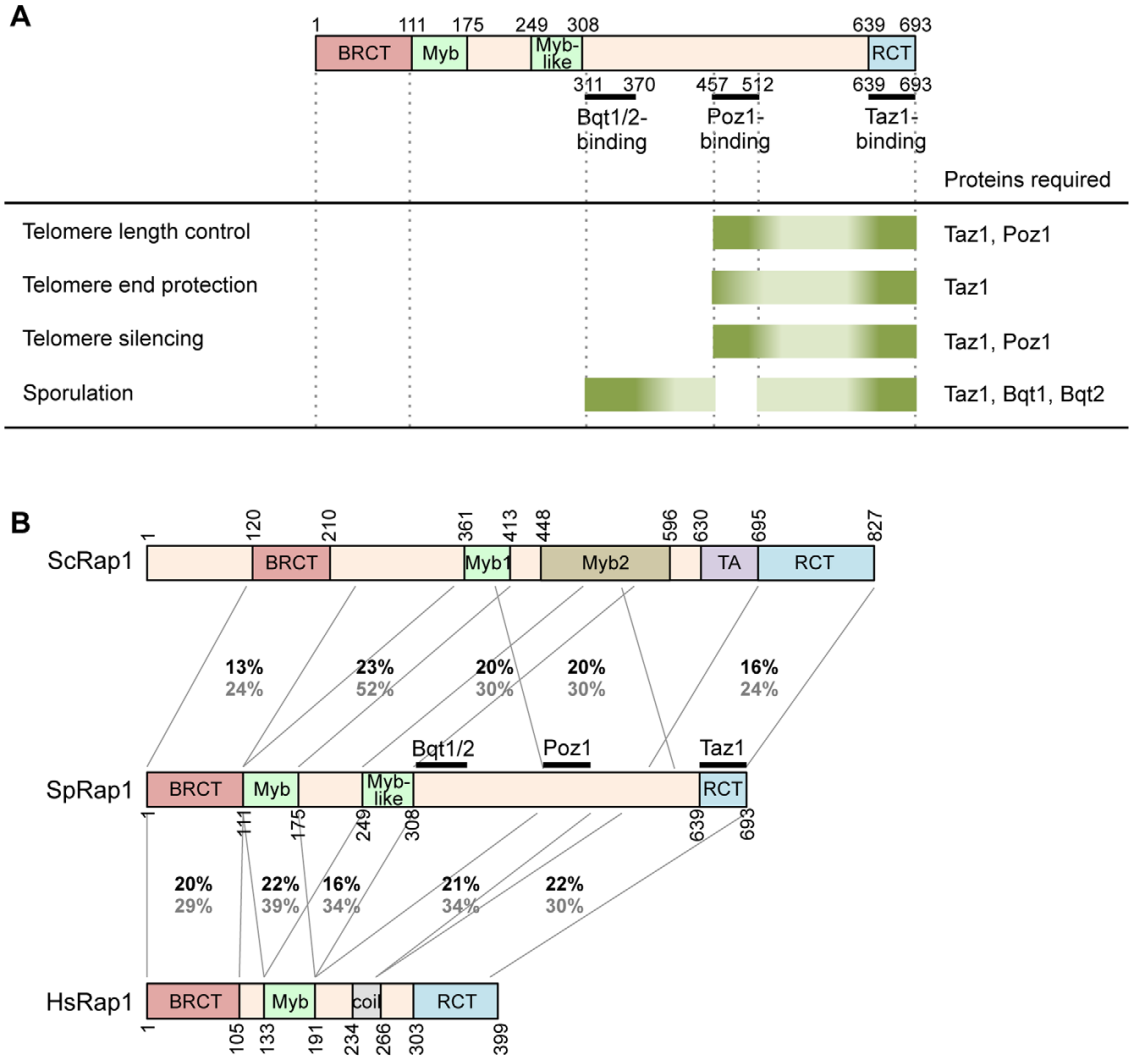
Summary of the functional domains of *S. pombe* Rap1. (A) The interaction domains of Rap1 with its partners are shown in the upper part. Dark green bars indicate the regions responsible for each telomere function. Pale green bars indicate the regions whose involvement in each telomere function is unclear. The Rap1-interactors required for each telomere function are indicated at the right. (B) Sequence similarity between *S. cerevisiae* Rap1 (ScRap1), *S. pombe* Rap1 (SpRap1), and human Rap1 (HsRap1). Identity (indicated in black) and similarity (indicated in grey) of the amino acid sequences between the homologous domains were analyzed by the ClustalW program.

In this study, we identified the Rap1 domains responsible for the interactions with its partners, Bqt1/Bqt2 and Poz1. Bqt1/Bqt2, Poz1, and Taz1 bind to the distinct domains of Rap1 in the C-terminal half, which was essential for the primary telomere functions, such as telomere length control, telomere protection, telomere gene silencing, and normal spore formation after meiosis. In contrast, the long N-terminal region was dispensable for those telomere functions (Figure 5A).

Our results indicated that *S. pombe* Rap1 has multiple modules for the protein-protein interactions. This implies the possibility that the Rap1 partners, Bqt1/Bqt2, Poz1, and Taz1, can bind to a single Rap1 molecule simultaneously. In contrast, our recent study has shown that the long middle region of Rap1, i.e., 220–607 a.a., that overlaps with the Bqt1/Bqt2- and Poz1-binding sites, is required for the interaction between Rap1 and Bqt4 [25]. It will be interesting to solve the three-dimensional crystal or solution structure of the Rap1 complex with its multiple partners.

Intriguingly, the *rap1-C, -D* and Δ*Pz* mutations, which probably weaken the interaction between Rap1 and Bqt4, do not confer a significant deficiency in spore formation to the cells, although the *bqt4*Δ strain shows a high frequency of abnormal spore formation because Bqt4 plays an important role in telomere clustering in meiosis [15] (Figure****4). These observations imply that the existence of Bqt4 at the nuclear envelope, not the interaction between Rap1 and Bqt4, is critical for the normal progression of meiosis.

We have previously shown that the structure of the RCT domain of Rap1 is evolutionarily conserved and it acts as a protein-protein interaction domain [19]. The RCT domains of fission yeast and mammalian Rap1 associate with the TRF family proteins, Taz1 and TRF2, respectively. In contrast, the RCT domain of budding yeast Rap1 interacts with Rif1/Rif2 and Sir3/Sir4 to regulate telomere DNA length and telomere gene silencing, respectively [21], [22], [23], [24]. Thus, the partner of the RCT domain is conserved in fission yeast and mammals, not in budding yeast. This study demonstrated that the *rap1-I655R* mutant shows significant defects in the telomere DNA length control and gene silencing of the subtelomeric *tlh* genes (Figures 3A and 3C). In budding yeast, the *rap1* mutants expressing the C-terminal truncated forms of Rap1 lacking the RCT domain show defects in the telomere DNA length control and the telomere gene silencing [27], [28]. Therefore, both the RCT domains in budding and fission yeasts regulate telomere DNA length and telomere gene silencing in common. As mammalian Rap1 regulates telomere DNA length and gene silencing at subtelomeres [29], it is possible that the RCT domain of mammalian Rap1 is also important for the two functions, although it is currently unclear.

Our results showed that the *rap1-G, rap1*-Δ*Pz,* and *poz1*Δ strains are defective in telomere gene silencing like in the *rap1*Δ strain. The highly elongated telomeres in these mutants contain an excess of Taz1 binding sequences, which probably leads to partially Taz1-deficient telomeres as previously suggested [30]. Because Taz1 acts as one of the establishment factors for telomere heterochromatin by recruiting Clr4, a methyltransferase for histone H3-K9, to telomeres [13], the low abundance of Taz1 at subtelomeres possibly causes a deficiency in telomere heterochromatin structure in the mutants with highly elongated telomeres.

In this study, we have identified Bqt1/Bqt2- and Poz1-binding domains in *S. pombe* Rap1. A partial deletion of the Bqt1/Bqt2-binding domain resulted in the abnormal spore formation like in the *bqt1*Δ and *bqt2*Δ mutants, indicating that the meiotic telomere clustering is mediated by the interaction between the Bqt1/Bqt2-binding site of Rap1 and Bqt1/Bqt2. Although meiotic chromosomal bouquet formation is conserved in eukaryotes, no obvious homologs of Bqt1 and Bqt2 are found in other organisms, with the exception of related fission yeast *Schizosaccharomyces japonicus* [31]. Consistently, the budding yeast and human Rap1 proteins lack a region homologous to the Bqt1/Bqt2-binding domain (Figure 5B), and the meiotic bouquet formation does not require Rap1 in mouse [32]. Therefore, the meiotic function of Rap1 may not be conserved in budding yeast and mammals. In contrast, we found that the region containing the Poz1-binding site of *S. pombe* Rap1 has a weak homology with the region containing the parts of Myb1 and Myb2 of *S. cerevisiae* Rap1 and with the region (192–259 a.a.) of human Rap1 (Figure 5B). This implies the possibility that the structure of the Poz1-binding domain is evolutionarily conserved and that human Rap1 interacts with a protein other than TRF2. In addition, the middle region of budding yeast Rap1 also has a weak homology to the Poz1-binding domain, although their structural and functional similarities are unclear.

It was reported that Taz1 is required for the prevention of genome-wide DNA double-strand breaks (DSBs), and the *taz1*Δ cells accumulate DSBs and display growth defect at the cold temperature of 20°C [33]. Furthermore, the *taz1*Δ mutant is hypersensitive to DNA damage induced by the MMS (methyl methanesulfonate) treatment [33]. We found no obvious defect in the cell growth of any *rap1* mutant at 20°C and at 36°C or in the presence of MMS (Figure S2), whereas the *taz1*Δ mutant showed a weak sensitivity to MMS, suggesting that Rap1 does not play a major role in the responses to temperature stress and to DNA damage. Recently, Tazumi *et al*. reported that Taz1 is localized near late origins and regulates the timing of replication [34], implying that Rap1 is also involved in the regulation of replication timing through binding to replication origins via Taz1.

The following question has been raised from our results: what are the roles of the BRCT and Myb domains of Rap1? The budding yeast Rap1 directly interacts with telomere DNA and other DNA targets, and the two Myb domains of Rap1 are necessary for its binding to DNA [20]. A previous report has indicated that the BRCT domain of human Rap1 contributes to the heterogeneity of the telomere DNA length [35]. The major roles of the BRCT and Myb domains of human Rap1, however, are currently unknown. Recently, mammalian Rap1 has been shown to bind to non-telomeric chromosomal DNA to regulate the expression of various genes and does not necessarily require TRF2 for its binding to non-telomeric loci [29]. The BRCT domains are also known to mediate various activities: protein-protein interactions, DNA-binding, and phosphate-binding [36]. Thus, the human Rap1 may directly bind to non-telomeric DNA through its BRCT and/or Myb domains, and thus *S. pombe* Rap1 may also bind to non-telomeric DNA via its BRCT and/or Myb domains, although whether the fission yeast Rap1 is localized at non-telomeric chromosomal regions is currently unknown. Further analyses will be required to uncover the functions of the N-terminal region of Rap1.

## Materials and Methods

### Strains and General Techniques for *S. pombe*

The *S. pombe* strains used in this study are listed in Table 1. The YES, MEA, and EMM media were used for cell culturing. The growth media, basic genetics, and biochemical techniques have been previously described [37]–[38]. For the deletions of the *rap1* ^+^, *taz1* ^+^, *poz1* ^+^, *bqt1* ^+^, *bqt2* ^+^, and *bqt4* ^+^ genes, each open reading frame (ORF) was replaced with the *ura4* ^+^, *kanMX6, or hphMX6* cassette by homologous recombination [39]. For the generation of the *rap1* deletion mutants, *rap1*-deleted cells (*rap1*::*ura4* ^+^) were transformed with the DNA fragments containing each truncated form of the *rap1* ^+^ ORF, and the transformants were selected on YES plates containing 5-fluoroorotic acid (5-FOA). The replacement of chromosomal DNA was confirmed by PCR and DNA sequencing. Because FOA can induce mutations in the mitochondrial and nuclear DNA, the cell growth on non-fermentable carbon source media YEEG (0.5% yeast extract, 3% ethanol, 3% glycerol) and YEG (0.5% yeast extract, 3% glycerol, 0.1% glucose) was examined to confirm that the mitochondrial functions are normal (Figure S3).

**Table 1:** *S. pombe* strains used in this study.

Fig. 1	JK3068	*h^–^ lys1^+^*::*nmt1-GFP-bqt1 aur1* ^r^::*nmt1-bqt2-myc bqt1*::*LEU2 bqt2*::*ura4* ^+^ *ade6-M216 leu1.32 ura4-D18*
	JK2980	*h^90^ poz1-3Flag*:*hph taz1-3HA*:*ura4* ^+^ *ade6-M210 leu1.32 ura4-D18*
Fig. 2, 3A, 3B	JK317	*h^–^ leu1.32 ura4-D18*
	IF1814	*h^–^ rap1*::*hph leu1.32 ura4-D18*
	JP1903	*h^–^ rap1-*Δ*2–110* (*rap1-A*) *leu1.32 ura4-D18*
	JP1907	*h^–^ rap1-Δ2–174* (*rap1-B*) *leu1.32 ura4-D18*
	JP1911	*h^–^ rap1-Δ2–248* (*rap1-C*) *leu1.32 ura4-D18*
	JP1915	*h^–^ rap1-Δ2–300* (*rap1-D*) *leu1.32 ura4-D18*
	JP1919	*h^–^ rap1-Δ2–370* (*rap1-E*) *leu1.32 ura4-D18*
	JP1922	*h^–^ rap1-Δ2–456* (*rap1-F*) *leu1.32 ura4-D18*
	JP1926	*h^–^ rap1-Δ2–512* (*rap1-G*) *leu1.32 ura4-D18*
	JP408	*h^–^ rap1-I655R leu1.32 ura4-D18*
	JP1856	*h^–^ rap1-Δ457–512* (*rap1-ΔPoz1-binding site*) *leu1.32 ura4-D18*
	IF1967	*h^–^ rap1-Δ341–370* (*rap1-ΔBqt1/2-binding site*) *leu1.32 ura4-D18*
	JK702	*h^–^ taz1*::*ura4* ^+^ *leu1.32 ura4-D18*
	JP795	*h^–^ poz1*::*ura4* ^+^ *leu1.32 ura4-D18*
	MT1806	*h^–^ bqt1*::*hph leu1.32 ura4-D18*
	MT1808	*h^–^ bqt2*::*hph leu1.32 ura4-D18*
Fig. 3C, 4, S1, S2, S3	JP245	*h^90^ ade6-M210 leu1.32 ura4-D18*
Fig. 3C, S1, S2, S3	IF1972	*h^90^ rap1-Δ1–693* (*rap1Δ*) *ade6-M210 leu1.32 ura4-D18*
	JP1906	*h^90^ rap1-Δ2–110* (*rap1-A*) *ade6-M210 leu1.32 ura4-D18*
	JP1910	*h^90^ rap1-Δ2–174* (*rap1-B*) *ade6-M210 leu1.32 ura4-D18*
	JP1914	*h^90^ rap1-Δ2–248* (*rap1-C*) *ade6-M210 leu1.32 ura4-D18*
	JP1918	*h^90^ rap1-Δ2–300* (*rap1-D*) *ade6-M210 leu1.32 ura4-D18*
	JP1921	*h^90^ rap1-Δ2–370* (*rap1-E*) *ade6-M210 leu1.32 ura4-D18*
	JP1925	*h^90^ rap1-Δ2–456* (*rap1-F*) *ade6-M210 leu1.32 ura4-D18*
	JP1929	*h^90^ rap1-Δ2–512* (*rap1-G*) *ade6-M210 leu1.32 ura4-D18*
	IF1974	*h^90^ rap1-I655R ade6-M210 leu1.32 ura4-D18*
	IF1859	*h^90^ rap1-Δ457–512* (*rap1-ΔPoz1-binding site*) *ade6-M210 leu1.32 ura4-D18*
	IF1970	*h^90^ rap1-Δ341-370* (*rap1-ΔBqt1/2-binding site*) *ade6-M210 leu1.32 ura4-D18*
Fig. 3C, 4, S2	TN266	*h^90^ taz1*::*ura4* ^+^ *ade6-M210 leu1.32 ura4-D18*
	JK3062	*h^90^ poz1*::*hph ade6-M210 leu1.32 ura4-D18*
	MT1671	*h^90^ bqt1*::*hph ade6-M210 leu1.32 ura4-D18*
	MT1673	*h^90^ bqt2*::*hph ade6-M210 leu1.32 ura4-D18*
Fig. 4	IF1971	*h^90^ rap1-Δ1–693* (*rap1Δ*) *ade6-M210 leu1.32 ura4-D18*
	JP1905	*h^90^ rap1-Δ2–110* (*rap1-A*) *ade6-M210 leu1.32 ura4-D18*
	JP1909	*h^90^ rap1-Δ2–174* (*rap1-B*) *ade6-M210 leu1.32 ura4-D18*
	JP1913	*h^90^ rap1-Δ2–248* (*rap1-C*) *ade6-M210 leu1.32 ura4-D18*
	JP1917	*h^90^ rap1-Δ2–300* (*rap1-D*) *ade6-M210 leu1.32 ura4-D18*
	JP1920	*h^90^ rap1-Δ2–370* (*rap1-E*) *ade6-M210 leu1.32 ura4-D18*
	JP1924	*h^90^ rap1-Δ2–456* (*rap1-F*) *ade6-M210 leu1.32 ura4-D18*
	JP1928	*h^90^ rap1-Δ2–512* (*rap1-G*) *ade6-M210 leu1.32 ura4-D18*
	IF1973	*h^90^ rap1-I655R ade6-M210 leu1.32 ura4-D18*
	IF1858	*h^90^ rap1-Δ457-512* (*rap1-ΔPoz1-binding site*) *ade6-M210 leu1.32 ura4-D18*
	IF1969	*h^90^ rap1-Δ341-370* (*rap1-ΔBqt1/2-binding site*) *ade6-M210 leu1.32 ura4-D18*
	JP839	*h^90^ bqt4*::*ura4* ^+^ *ade6-M210 leu1.32 ura4-D18*
Fig. S2	JK325	*h^–^ rad3*::*ura4* ^+^ *leu1.32 ura4-D18*
	JK685	*h^–^ crb2*::*ura4* ^+^ *ade6-M210 leu1.32 ura4-D18*

### Pull-down Assays

Parts of the *rap1* ^+^ ORF were amplified by PCR and cloned into pGEX-5X-2 (GE Healthcare, Little Chalfont, UK). The *E. coli* BL21-CodonPlus (Stratagene, San Diego, USA) was transformed with each plasmid, and each glutathione *S*-transferase (GST)-Rap1 fusion protein was purified from bacteria using Glutathione Sepharose 4B (GE Healthcare). GST-Rap1 proteins (∼1 µg) bound to glutathione beads were mixed with *S. pombe* cell extracts and washed with TNE buffer (40****mM Tris-HCl [pH 7.5], 150****mM NaCl, 5 mM EDTA, 50 mM NaF, 20 mM ß-glycerophosphate). The protein complexes were boiled in SDS sample buffer and analyzed by SDS-PAGE, followed by western blotting.

### Yeast Two- and Three-hybrid Assays

A series of truncated forms of the *rap1* ^+^ gene was amplified by PCR and cloned into pACT2 (prey; Clontech, Mountain View, USA). The entire ORFs of *taz1* ^+^ and *poz1* ^+^ were amplified by PCR and individually cloned into pGBKT7 (bait; Clontech) for the two-hybrid assays. To detect the interactions between Rap1 and Bqt1/Bqt2 in the three-hybrid assays, pBridge-*bqt1* ^+^/*bqt2* ^+^ [17] (bait), which expresses the Gal4-BD-Bqt1 and Bqt2 proteins, was used. The *S. cerevisiae* strain Y190 (*MATa*, *ura3–52*, *his3*-Δ*200*, *lys2*–*801*, *ade2–101*, *trp1–901*, *leu2–3*, *112*, *gal4*Δ, *gal80*Δ, *LYS2*::*GAL1_UAS_*-*HIS3_TATA_*-*HIS3*, *URA3*::*GAL1_UAS_*-*GAL1_TATA_*-*lazZ*, *cyh* ^r^ *2*) was transformed with each plasmid and assayed for ß-galactosidase activity according to the manufacturer's instructions.

### Antibodies

For the production of polyclonal antibodies against the C-terminal region of Rap1, the bacterial GST-Rap1 (370–693 a.a.) protein was used to immunize rabbits (Medical & Biological Laboratories, Nagoya, Japan), and the antibodies were affinity purified using GST-Rap1 (370–693 a.a.) protein on Immobilon P transfer membranes (Millipore, Billerica, USA). Anti-Myc (9E10; Santa Cruz Biotech., Santa Cruz, USA), anti-Flag (F3165; Sigma, St. Louis, USA), anti-HA (16B12; Covance, Princeton, USA), and anti-PSTAIR (P7962; Sigma) antibodies were used to detect Bqt2-Myc, Poz1-Flag, Taz1-HA, and Cdc2, respectively.

### Phosphatase Treatment of the Rap1 Protein

Rap1 proteins were immunoprecipitated from cell extracts of 50-ml cultures using anti-Rap1. The immunoprecipitates were washed with CIAP buffer (50 mM Tris-HCl [pH 9.0], 1 mM MgCl_2_) twice and treated with 60 U of CIAP (Takara, Otsu, Japan) in the same buffer for 1 hour at 30°C with or without phosphatase inhibitors (50 mM NaF, 1****mM NaVO_4_, 10 mM EGTA, 50 mM ß-glycerophosphate, 1× PhosSTOP [Roche, Basel, Switzerland]). The immunoprecipitates were washed twice with MASS buffer (25 mM HEPES-KOH [pH 7.5], 200****mM NaCl, 10% glycerol, 0.1% NP-40) with or without phosphatase inhibitors and analyzed by immunoblotting using anti-Rap1.

### Telomere Southern Blot Analysis

Genomic DNA (20 µg) was digested with *Eco*RI and analyzed on Hybond N^+^ nylon membranes (GE Healthcare) as described [12]. The *Apa*1-*Eco*RI fragment of pAMP1 [40], which contains the ∼300 bp of telomere repeats, was used as a probe.

### Pulse-field Gel Electrophoresis (PFGE)

For the detection of telomere end fusions, PFGE was performed as described previously [19].

### Reverse Transcription-Quantitative PCR

Total RNA was extracted from each strain and treated with recombinant DNase I (Takara). cDNA was generated using 5 µg of RNA as a template with High Capacity cDNA Reverse Transcription Kit (Applied Biosystems, Carlsbad, USA) and quantified with StepOne^TM^ Real-Time PCR System (Applied Biosystems) and Fast SYBR® Green Master Mix (Applied Biosystems). The following primer sets were used to detect cDNA: for *tlh-dh*, 5′-TCGTCTTGTAGCAGCATGTGA-3′ and 5′-GAGATGAACGTATCTCTATCGAC-3′ (as previously described [26]); for *his1* ^+^, 5′- CGAAGACGTGCTTCAGCGA-3′ and 5′-TGTCCACCTCGGAATCACTG-3′.

### Spore Formation Assays

Cells were grown on YES plates and then were grown on MEA plates at 28°C for 2****days. The spore formation images were taken with a BX53 upright microscope (Olympus, Tokyo, Japan).

## Supporting Information

Figure S1 **Reproduction of** **Figure 3A** **and** **Figure 4B** **.** (A) Telomere DNA length was analyzed using other strains carrying the same *rap1* alleles as those used in Figure 3A. Telomere southern blot was performed as in Figure 3A. (B) Spore formation was analyzed using other homothallic strains carrying the same *rap1* alleles as those used in Figure 3C. *n*>200 for each strain.
(TIF)

Figure S2 **The** ***rap1*** **mutants are not sensitive to high and low temperatures and to MMS.** (A) Serial cell dilutions of each strain were spotted on YES plates and incubated at 36°C and at 20°C. (B) Serial cell dilutions of each strain were spotted on YES plates containing without or with 0.003–0.01% MMS and incubated at 32°C. The *rad3*Δ and *crb2*Δ mutants are the positive controls for the MMS-sensitive strains.
(TIF)

Figure S3 **Normal cell growth of the** ***rap1*** **mutants in the non-fermentable condition.** Serial cell dilutions of each strain were spotted on YES, YEG (3% glycerol, 0.1% glucose), and YEEG (3% ethanol, 3% glycerol) and incubated at 32°C.
(TIF)

## References

[1] Meyne J, Ratliff RL, Moyzis RK (1989) Conservation of the human telomere sequence (TTAGGG)n among vertebrates. Proc Nat Acad Sci USA 86: 7049–7053. 2780561 10.1073/pnas.86.18.7049 PMC297991

[2] Hiraoka Y, Henderson E, Blackburn E (1998) Not so peculiar: fission yeast telomere repeats. Trends Biochem Sci 23: 126. 9584612 10.1016/s0968-0004(98)01176-1

[3] Kanoh J, Ishikawa F (2003) Composition and conservation of the telomeric complex. Cell Mol Life Sci 60: 2295–2302. 14625676 10.1007/s00018-003-3245-y PMC11138895

[4] Smogorzewska A, de Lange T (2004) Regulation of telomerase by telomeric proteins. Annu Rev Biochem 73: 177–208. 15189140 10.1146/annurev.biochem.73.071403.160049

[5] Ferreira MG, Miller KM, Cooper JP (2004) Indecent exposure: when telomeres become uncapped. Mol Cell 13: 7–18. 14731390 10.1016/s1097-2765(03)00531-8

[6] Stewart JA, Chaiken MF, Wang F, Price CM (2012) Maintaining the end: Roles of telomere proteins in end-protection, telomere replication and length regulation. Mut Res 730: 12–19. 21945241 10.1016/j.mrfmmm.2011.08.011 PMC3256267

[7] de La Roche Saint-Andre C (2008) Alternative ends: telomeres and meiosis. Biochimie 90: 181–189. 17905509 10.1016/j.biochi.2007.08.010

[8] Cooper JP, Nimmo ER, Allshire RC, Cech TR (1997) Regulation of telomere length and function by a Myb-domain protein in fission yeast. Nature 385: 744–747. 9034194 10.1038/385744a0

[9] Cooper JP, Watanabe Y, Nurse P (1998) Fission yeast Taz1 protein is required for meiotic telomere clustering and recombination. Nature 392: 828–831. 9572143 10.1038/33947

[10] Chikashige Y, Hiraoka Y (2001) Telomere binding of the Rap1 protein is required for meiosis in fission yeast. Curr Biol 11: 1618–1623. 11676924 10.1016/s0960-9822(01)00457-2

[11] Ferreira MG, Cooper JP (2001) The fission yeast Taz1 protein protects chromosomes from Ku-dependent end-to-end fusions. Mol Cell 7: 55–63. 11172711 10.1016/s1097-2765(01)00154-x

[12] Kanoh J, Ishikawa F (2001) spRap1 and spRif1, recruited to telomeres by Taz1, are essential for telomere function in fission yeast. Curr Biol 11: 1624–1630. 11676925 10.1016/s0960-9822(01)00503-6

[13] Kanoh J, Sadaie M, Urano T, Ishikawa F (2005) Telomere binding protein Taz1 establishes Swi6 heterochromatin independently of RNAi at telomeres. Curr Biol 15: 1808–1819. 16243027 10.1016/j.cub.2005.09.041

[14] Miller KM, Ferreira MG, Cooper JP (2005) Taz1, Rap1 and Rif1 act both interdependently and independently to maintain telomeres. EMBO J 24: 3128–3135. 16096639 10.1038/sj.emboj.7600779 PMC1201358

[15] Chikashige Y, Yamane M, Okamasa K, Tsutsumi C, Kojidani T, et al (2009) Membrane proteins Bqt3 and −4 anchor telomeres to the nuclear envelope to ensure chromosomal bouquet formation. J Cell Biol 187: 413–427. 19948484 10.1083/jcb.200902122 PMC2779253

[16] Bianchi A, Shore D (2008) How telomerase reaches its end: mechanism of telomerase regulation by the telomeric complex. Mol Cell 31: 153–165. 18657499 10.1016/j.molcel.2008.06.013

[17] Chikashige Y, Tsutsumi C, Yamane M, Okamasa K, Haraguchi T, et al (2006) Meiotic proteins Bqt1 and Bqt2 tether telomeres to form the bouquet arrangement of chromosomes. Cell 125: 59–69. 16615890 10.1016/j.cell.2006.01.048

[18] Miyoshi T, Kanoh J, Saito M, Ishikawa F (2008) Fission yeast Pot1-Tpp1 protects telomeres and regulates telomere length. Science 320: 1341–1344. 18535244 10.1126/science.1154819

[19] Chen Y, Rai R, Zhou ZR, Kanoh J, Ribeyre C, et al (2011) A conserved motif within RAP1 has diversified roles in telomere protection and regulation in different organisms. Nat Struct Mol Biol 18: 213–221. 21217703 10.1038/nsmb.1974 PMC3688267

[20] Konig P, Giraldo R, Chapman L, Rhodes D (1996) The crystal structure of the DNA-binding domain of yeast RAP1 in complex with telomeric DNA. Cell 85: 125–136. 8620531 10.1016/s0092-8674(00)81088-0

[21] Hardy CF, Sussel L, Shore D (1992) A RAP1-interacting protein involved in transcriptional silencing and telomere length regulation. Genes Dev 6: 801–814. 1577274 10.1101/gad.6.5.801

[22] Moretti P, Freeman K, Coodly L, Shore D (1994) Evidence that a complex of SIR proteins interacts with the silencer and telomere-binding protein RAP1. Genes Dev 8: 2257–2269. 7958893 10.1101/gad.8.19.2257

[23] Cockell M, Palladino F, Laroche T, Kyrion G, Liu C, et al (1995) The carboxy termini of Sir4 and Rap1 affect Sir3 localization: evidence for a multicomponent complex required for yeast telomeric silencing. J Cell Biol 129: 909–924. 7744964 10.1083/jcb.129.4.909 PMC2120499

[24] Wotton D, Shore D (1997) A novel Rap1p-interacting factor, Rif2p, cooperates with Rif1p to regulate telomere length in *Saccharomyces cerevisiae* . Genes Dev 11: 748–760. 9087429 10.1101/gad.11.6.748

[25] Fujita I, Nishihara Y, Tanaka M, Tsujii H, Chikashige Y, et al. (2012) Telomere-nuclear envelope dissociation promoted by Rap1 phosphorylation ensures faithful chromosome segregation. Curr Biol. Available: http://dx.doi.org/10.1016/j.cub.2012.1008.1019. 10.1016/j.cub.2012.08.019 22959349

[26] Hansen KR, Ibarra PT, Thon G (2006) Evolutionary-conserved telomere-linked helicase genes of fission yeast are repressed by silencing factors, RNAi components and the telomere-binding protein Taz1. Nucleic Acids Res 34: 78–88. 16407326 10.1093/nar/gkj415 PMC1326240

[27] Kyrion G, Boakye KA, Lustig AJ (1992) C-terminal truncation of RAP1 results in the deregulation of telomere size, stability, and function in *Saccharomyces cerevisiae.* . Mol Cell Biol 12: 5159–5173. 1406688 10.1128/mcb.12.11.5159-5173.1992 PMC360450

[28] Kyrion G, Liu K, Liu C, Lustig AJ (1993) RAP1 and telomere structure regulate telomere position effects in *Saccharomyces cerevisiae.* . Genes Dev 7: 1146–1159. 8319907 10.1101/gad.7.7a.1146

[29] Martinez P, Thanasoula M, Carlos AR, Gomez-Lopez G, Tejera AM, et al (2010) Mammalian Rap1 controls telomere function and gene expression through binding to telomeric and extratelomeric sites. Nat Cell Biol 12: 768–780. 20622869 10.1038/ncb2081 PMC3792482

[30] Dehe PM, Rog O, Ferreira MG, Greenwood J, Cooper JP (2012) Taz1 enforces cell-cycle regulation of telomere synthesis. Mol Cell 46: 797–808. 22633956 10.1016/j.molcel.2012.04.022

[31] Chikashige Y, Haraguchi T, Hiraoka Y (2007) Another way to move chromosomes. Chromosoma 116: 497–505. 17639451 10.1007/s00412-007-0114-8

[32] Scherthan H, Sfeir A, de Lange T (2011) Rap1-independent telomere attachment and bouquet formation in mammalian meiosis. Chromosoma 120: 151–157. 20927532 10.1007/s00412-010-0295-4 PMC3132479

[33] Miller KM, Cooper JP (2003) The telomere protein Taz1 is required to prevent and repair genomic DNA breaks. Mol Cell 11: 303–313. 12620220 10.1016/s1097-2765(03)00041-8

[34] Tazumi A, Fukuura M, Nakato R, Kishimoto A, Takenaka T, et al (2012) Telomere-binding protein Taz1 controls global replication timing through its localization near late replication origins in fission yeast. Genes & development 26: 2050–2062. 22987637 10.1101/gad.194282.112 PMC3444731

[35] Li B, de Lange T (2003) Rap1 affects the length and heterogeneity of human telomeres. Mol Biol Cell 14: 5060–5068. 14565979 10.1091/mbc.E03-06-0403 PMC284807

[36] Leung CC, Glover JN (2011) BRCT domains: easy as one, two, three. Cell Cycle 10: 2461–2470. 21734457 10.4161/cc.10.15.16312 PMC3180187

[37] Moreno S, Klar A, Nurse P (1991) Molecular genetic analysis of fission yeast *Schizosaccharomyces pombe* . Methods Enzymol 194: 795–823. 2005825 10.1016/0076-6879(91)94059-l

[38] Forsburg SL, Rhind N (2006) Basic methods for fission yeast. Yeast 23: 173–183. 16498704 10.1002/yea.1347 PMC5074380

[39] Bahler J, Wu JQ, Longtine MS, Shah NG, McKenzie A 3rd, et al (1998) Heterologous modules for efficient and versatile PCR-based gene targeting in *Schizosaccharomyces pombe* . Yeast 14: 943–951. 9717240 10.1002/(SICI)1097-0061(199807)14:10<943::AID-YEA292>3.0.CO;2-Y

[40] Matsuura A, Naito T, Ishikawa F (1999) Genetic control of telomere integrity in *Schizosaccharomyces pombe*: *rad3* ^+^ and *tel1* ^+^ are parts of two regulatory networks independent of the downstream protein kinases *chk1* ^+^ and *cds1* ^+^ . Genetics 152: 1501–1512. 10430579 10.1093/genetics/152.4.1501 PMC1460706

