# Supplementary figures and images for: Identification of the Functional Domains of the Telomere Protein Rap1 in Schizosaccharomyces pombe
Source: PLoS One. 2012 Nov 2;7(11):e49151. doi: 10.1371/journal.pone.0049151 (PMC3487762; doi:10.1371/journal.pone.0049151)

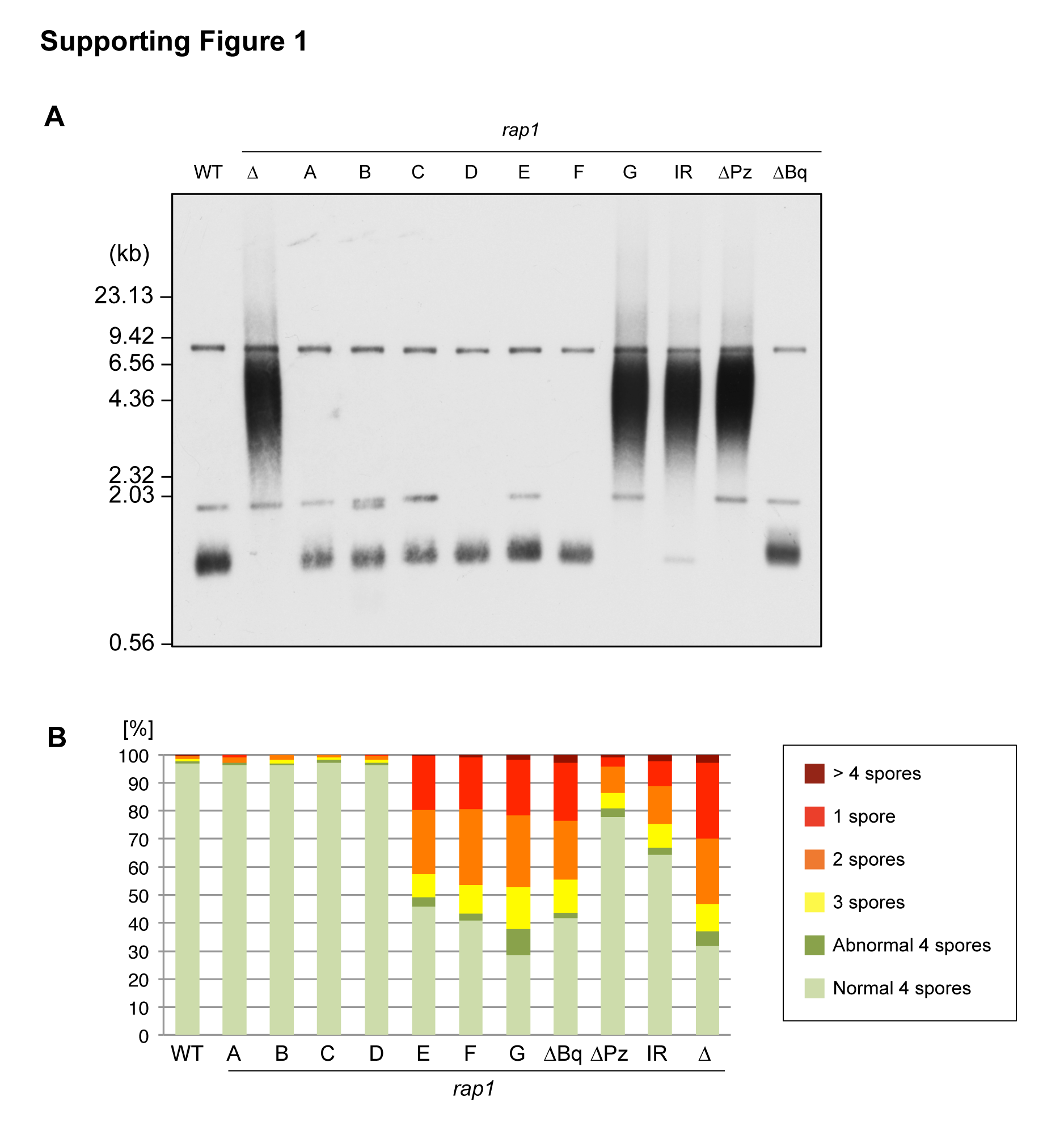

Supplement: Figure S1 — Reproduction of Figure 3A and Figure 4B . (A) Telomere DNA length was analyzed using other strains carrying the same rap1 alleles as those used in Figure 3A. Telomere southern blot was performed as in Figure 3A. (B) Spore formation was analyzed using other homothallic strains carrying the same rap1 alleles as those used in Figure 3C. n>200 for each strain. (TIF) [file pone.0049151.s001.tif]

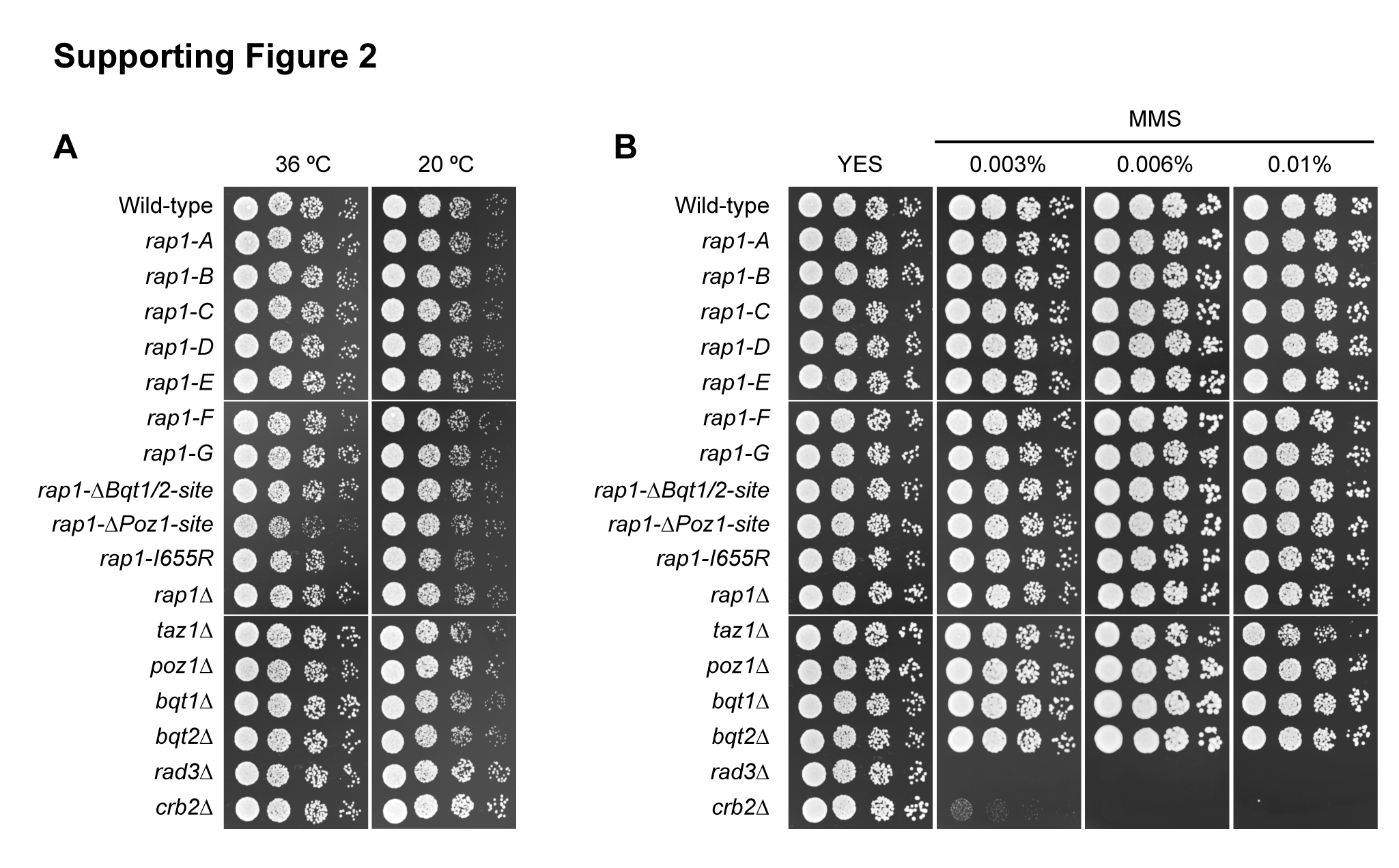

Supplement: Figure S2 — The rap1 mutants are not sensitive to high and low temperatures and to MMS. (A) Serial cell dilutions of each strain were spotted on YES plates and incubated at 36°C and at 20°C. (B) Serial cell dilutions of each strain were spotted on YES plates containing without or with 0.003–0.01% MMS and incubated at 32°C. The rad3Δ and crb2Δ mutants are the positive controls for the MMS-sensitive strains. (TIF) [file pone.0049151.s002.tif]

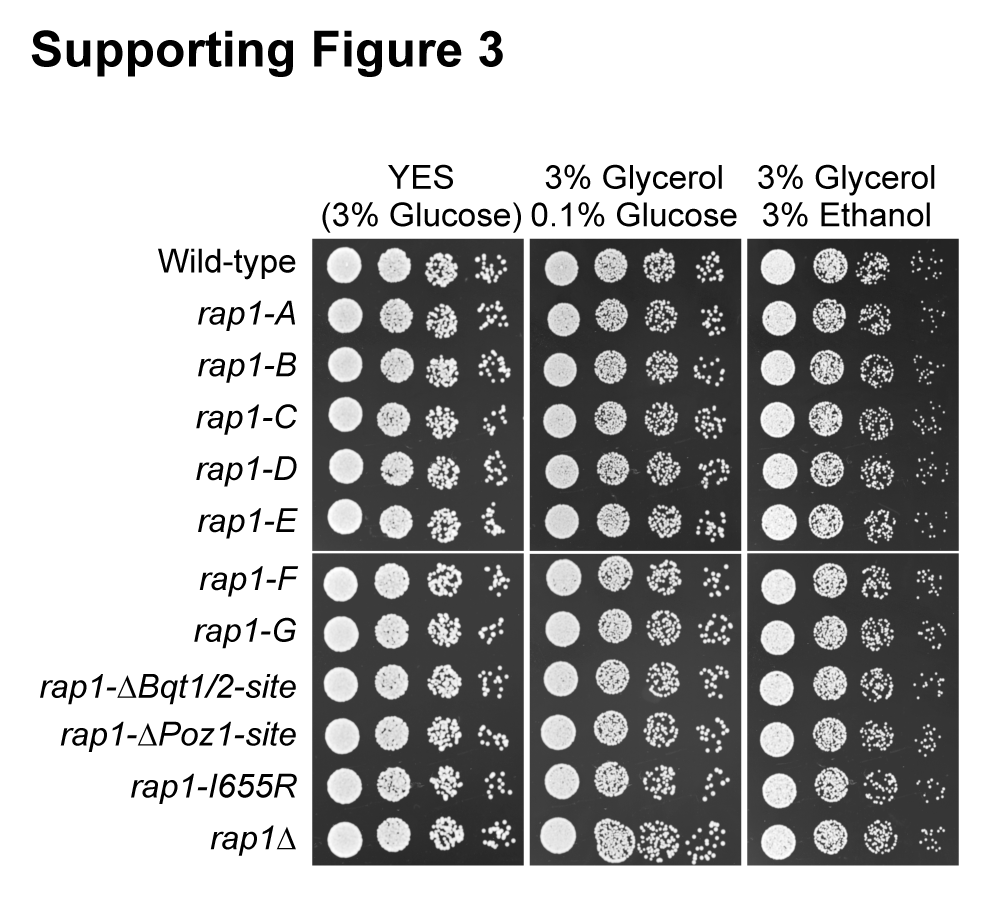

Supplement: Figure S3 — Normal cell growth of the rap1 mutants in the non-fermentable condition. Serial cell dilutions of each strain were spotted on YES, YEG (3% glycerol, 0.1% glucose), and YEEG (3% ethanol, 3% glycerol) and incubated at 32°C. (TIF) [file pone.0049151.s003.tif]
